# A Pediatric Case of Stevens-Johnson Syndrome With Extensive Palmoplantar Involvement Following Varicella Infection

**DOI:** 10.7759/cureus.108300

**Published:** 2026-05-05

**Authors:** Adil A Alsweis, Shatha B Omar, Ola B Omar, Amr Khaled, Mohammad Hamdan

**Affiliations:** 1 Department of Medicine, An-Najah National University, Nablus, PSE; 2 Department of Pediatric Allergy and Immunology, Ministry of Health, Nablus, PSE

**Keywords:** acute mucocutaneous eruption, ibuprofen, pediatric sjs, stevens-johnson syndrome, varicella infection

## Abstract

Stevens-Johnson syndrome (SJS) is a rare but dangerous clinical condition characterized by extensive skin lesions and erosive mucosal lesions. SJS is typically caused by an allergic reaction to a medicine, and occasionally, infectious causes can provoke the syndrome. The illness is considered a medical emergency, and treatment varies based on symptoms, with a focus on lesion treatment, airway and hemodynamic stability, and opportunistic infection avoidance. We report a case of 10 years old female patient who presented with chickenpox accompanied by high-grade fever. Ibuprofen was used to relieve her fever, and then she experienced a worsening skin rash, mucosal ulcers, dysphagia, and eye symptoms. SJS was compatible with clinical findings. The temporal correlation raises the possibility of a medication-related side effect triggered during the course of viral infection or by the viral infection itself. Topical and oral corticosteroids were used to treat the patient, and the patient's symptoms significantly improved. SJS is a condition that requires multidisciplinary care and management. Our case demonstrates the possibility of SJS being caused by varicella zoster infection or after taking ibuprofen while having chickenpox. It highlights the significance of identifying warning symptoms early on, such as rash and mucosal involvement, and using drugs cautiously during viral diseases. Favorable results and the avoidance of serious complications can be achieved by promptly stopping the offending medication if present, and starting the proper therapy, including corticosteroids.

## Introduction

Stevens-Johnson syndrome (SJS) is an acute, rare, and potentially life-threatening severe cutaneous adverse reaction (SCAR) characterized by systemic symptoms, epidermal necrosis, and mucosal involvement. Mucosal epithelial necrosis may affect the lips, oral cavity, oropharynx, respiratory tract, gastrointestinal tract, and genitalia, leading to erosions, ulcerations, and epithelial detachment [1]. SJS is considered a delayed-type, T-cell-mediated hypersensitivity reaction [2]. The reported incidence of SJS/toxic epidermal necrolysis (TEN) ranges from approximately 1.2 to 7.4 cases per 1,000,000 persons annually; however, the exact incidence in the pediatric population remains uncertain [3].

TEN and SJS are considered part of the same disease spectrum within the group of SCARs [1]. Classification is based on the percentage of body surface area (BSA) with epidermal detachment: SJS involves less than 10% of BSA, SJS/TEN overlap involves 10%-30%, and TEN involves more than 30% [1,4]. Clinically, SJS/TEN is characterized by the rapid development of erythematous or purpuric macules, atypical targetoid lesions, painful mucosal erosions, and epidermal detachment. The rash is preceded by a prodromal phase of influenza-like symptoms, including fever and malaise [5]. Extensive keratinocyte death in the epidermis, mediated through apoptotic and necroptotic pathways, is the pathophysiological hallmark of the disease [2].

Erythema multiforme major (EMM) is a distinct clinical entity that should be differentiated from SJS/TEN, although overlap in clinical presentation may make distinction difficult. Classic target lesions with three concentric zones are more typical of EMM, whereas SJS/TEN more commonly presents with atypical targetoid lesions and widespread epidermal detachment. EMM is more often associated with infections, particularly respiratory infections, and is generally seen in younger patients, while SJS/TEN is more commonly associated with drug exposure [1].

Most of the time, drugs are thought to be the primary cause of SJS, which is the causative agent in over 80% of cases, especially with antibiotics and antipyretic analgesics [1,2]; however, infections with the Herpes simplex virus and *Mycoplasma pneumoniae* are well-established causes, along with a small number of cases where the etiology is still unknown. Allopurinol, trimethoprim-sulfamethoxazole and other sulfonamide antibiotics, aminopenicillins, cephalosporin, quinolones, carbamazepine, phenytoin, phenobarbital, and nonsteroidal anti-inflammatory drugs (NSAIDs) of the oxicam type are among the medications that are high risk of causing SJS [1].

The diagnosis is according to clinical observation, histological analysis, and the exclusion of numerous differential diagnoses [5]. A multidisciplinary strategy is necessary for the acute care of SJS. It is necessary to stop using possibly harmful medications right away. It is crucial to refer patients as soon as possible to a suitable medical facility for specialized supportive care since the average reported mortality rate of SJS in severe cases is 15-30%, mostly due to acute phase multiorgan failure driven by sepsis [3,6]. Cyclosporine A, immunoglobulins, and systemic corticosteroids are the most commonly utilized therapies for SJS. However, their benefits remain controversial [7].

In this paper, we will discuss a case of a 10-year-old female patient who was recently diagnosed with chickenpox and presented to the emergency department with new concerns for high-grade fever, painful oral ulcers, sore throat, and widespread rash, which is characteristic of erythema multiform. During hospitalization, her rash is worsening, and eye pain is reported, so the diagnosis of SJS is made, taking into consideration the NSAID she took during her initial management course and the clinical worsening that occurred during that. Since SJS is a rare but dangerous disorder that needs to be identified and treated effectively, this case highlights the need for pattern recognition of this condition. The most common cause of the reaction is medicine, but diagnosis by complete history and physical examination is required.

## Case presentation

A 10-year-old previously healthy girl was brought to the hospital by her mother with fever, progressive skin rash, and mucocutaneous involvement. She was in her usual state of health 20 days prior to admission when she began to develop a pruritic vesicular rash over her trunk and extremities. She was examined at the time of presentation and diagnosed with chickenpox. Her treatment was limited to topical calamine lotion and cetirizine 5 mg twice daily. She also had a high fever up to 39°C, for which she received ibuprofen syrup as needed for the first five days, with slight improvement. The vesicular lesions resolved within 14 days without complications. Fifteen days later, she again developed sudden, intense, nocturnal pruritus, and she developed a new eruption involving the mouth and extremities, especially elbows, knees, and both hands and feet. The eruption rapidly spread over two days, becoming widespread with confluent lesions and tense blisters surrounded by erythema, associated with intense itching and burning. She had a high fever again and developed arthralgia involving the wrists and knees, which limited her ability to move these joints. She denied photophobia, phonophobia, neck stiffness, vomiting, diarrhea, respiratory symptoms, urinary symptoms, gastrointestinal complaints, or recent contact with sick individuals.

At home, she was given ibuprofen 400 mg tablets as needed and paracetamol syrup with little improvement. One day later, she was admitted with fever and worsening oral ulcers, reduced oral intake, and a new complaint of dysphagia. She denied photophobia, phonophobia, neck stiffness, vomiting, diarrhea, respiratory symptoms, urinary symptoms, or gastrointestinal complaints, and she had no recent contact with sick individuals.

The patient had a history of meningitis that occurred three years ago; she had no chronic medical disease, no autoimmune disease, no known allergies, no previous surgical operations, and no chronic medications. She had age-appropriate milestones. She had been born at term by uncomplicated vaginal delivery, and the neonatal course was uneventful. She has been living with her parents and five healthy siblings. Her father had stopped smoking seven months ago, and her mother was a housewife. There was no family history of diabetes, hypertension, autoimmune disease, or any similar dermatologic disease. Immunization and nutritional history were age-appropriate.

**Figure 1:**
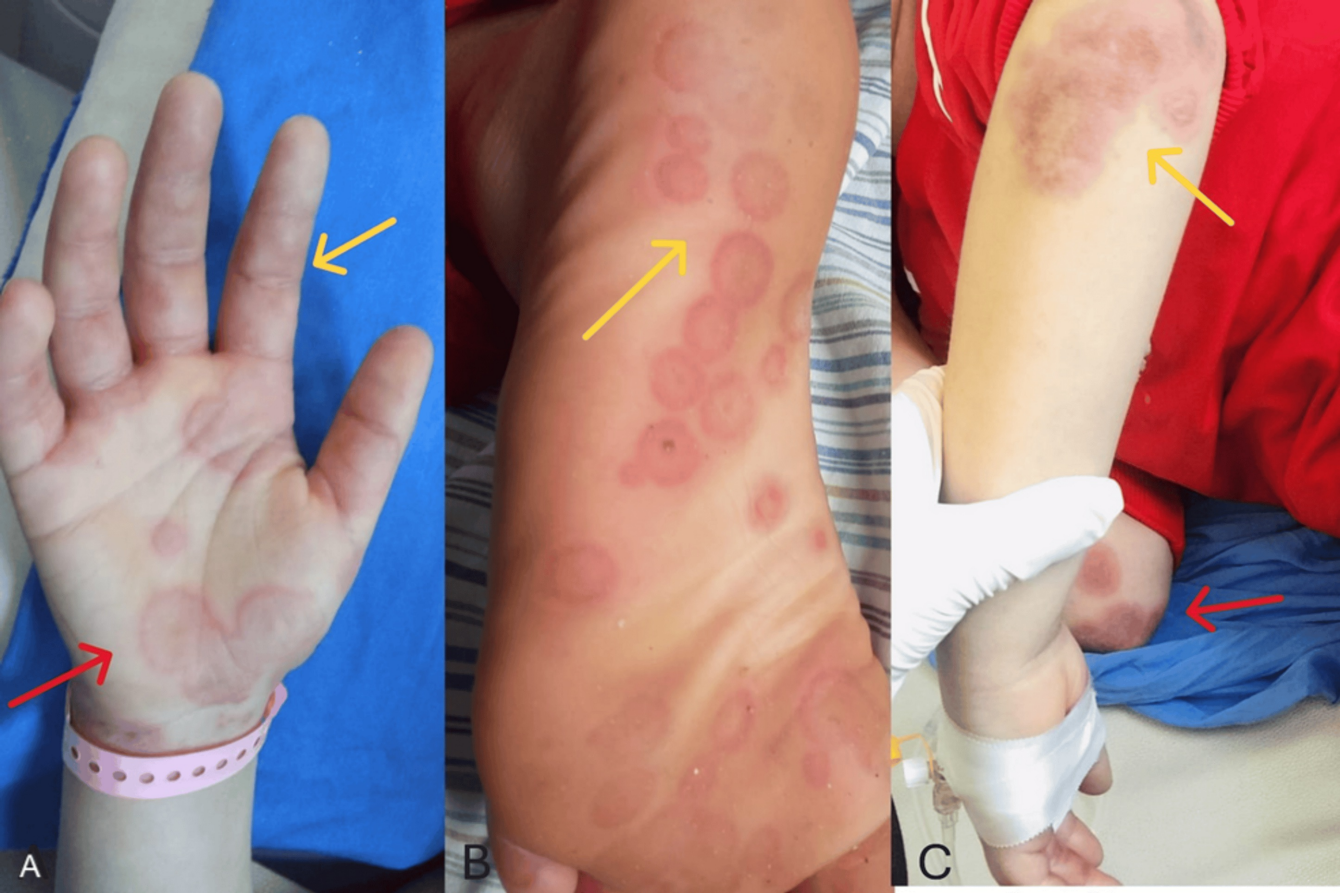
Cutaneous lesions involving the palms, soles, and extensor surfaces. (A) The yellow arrow indicates small, discrete erythematous palmar macules with central clearing; the red arrow indicates larger targetoid lesions on the lateral aspect of the wrist. (B) The yellow arrow indicates the confluent circular target lesions on the plantar aspect, highlighting areas of active inflammation. (C) The yellow arrow indicates a violaceous plaque with central dusky hyperpigmentation on the left extensor upper arm; the red arrow indicates other satellite targetoid lesions on the right forearm.

On examination at admission, she was mildly dehydrated but alert, with good air entry bilaterally and no added lung sounds. Her abdomen was soft and non-tender, with no hepatosplenomegaly. Neurological examination showed no meningeal signs. Dermatologic examination revealed multiple erythematous, round to oval target lesions distributed symmetrically over the upper and lower extremities, especially involving the extensor surfaces of the forearms, dorsum of the hands, lower legs, feet, and ankles, with positive Nikolsky's sign. The palms and soles were significantly involved, with palmar lesions appearing as small discrete erythematous macules with central clearing (Figure 1A), while plantar lesions were larger, more confluent, and forming irregular target plaques (Figure 1B), The extensor aspect of the forearm demonstrated a large, well-demarcated violaceous plaque with central hyperpigmentation, overlying dryness, and superficial scaling, surrounded by multiple satellite targetoid lesions, consistent with evolving post-inflammatory changes (Figure 1C). Musculoskeletal examination showed tenderness of both knees and limited movement of both knees and wrists due to pain.

**Figure 2:**
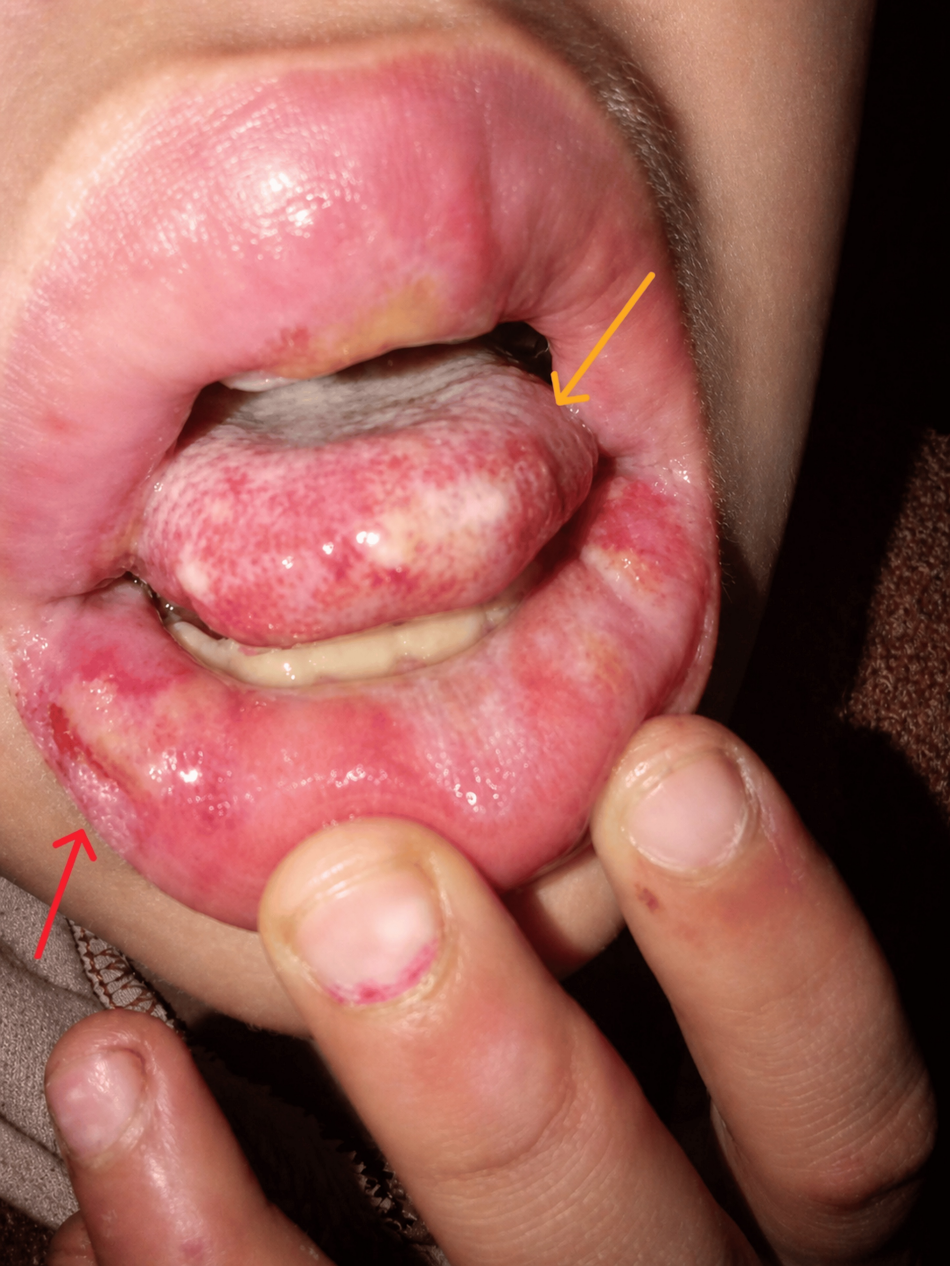
Oral and lip changes demonstrating erosions, ulcerations, and hemorrhagic crusting. The yellow arrow demonstrates a large superficial erosion on the inner lower lip with fibrinous exudate overlying it, consistent with active mucosal ulceration. The red arrow demonstrates peripheral fissuring and hemorrhagic crust formation along the vermilion border, consistent with the painful mucocutaneous breakdown associated with severe mucosal involvement in Stevens-Johnson syndrome.

Mucosal examination revealed significant oral involvement with diffuse erythema, multiple painful erosions, and shallow ulcerations affecting the lips and oral cavity. The lips were swollen and covered with hemorrhagic crusting along the vermilion border, with associated pain and difficulty in oral intake (Figure 2). The patient also developed eye itching around day 6 of admission without redness or discharge.

At admission, the initial clinical diagnosis was that of a post-varicella complication, including secondary bacterial skin infection, with *Pseudomonas aeruginosa* as a causative complicating organism. Other differential diagnoses were persistent or progressively viral infection, atypical infection (for example, *M. pneumoniae*), EMM, SJS, and immune-mediated or autoimmune mucocutaneous disease. Therefore, laboratory tests were carried out to assess markers of inflammation, hematologic status, renal and hepatic function, coagulation status, infectious agents, and immune-mediated or autoimmune causes.

Laboratory work was otherwise unremarkable (Table 1). Platelet and hemoglobin counts were normal with a mild decreasing trend. Differential count shift noted with an increase in lymphocyte percentage and a decrease in neutrophil percentage from admission to discharge, alongside clinical improvement. Partial clinical improvement was observed, with inflammatory markers showing a low C-reactive protein (CRP) level and persistently elevated erythrocyte sedimentation rate (ESR). Renal and liver function tests remained within normal limits throughout the hospital stay.

**Table 1:** Quantitative laboratory investigations with reference intervals and changes during hospitalization. CRP: C-reactive protein; ESR: erythrocyte sedimentation rate; BUN: blood urea nitrogen; AST: aspartate aminotransferase; ALT: alanine aminotransferase

Parameter	Admission	Discharge	Reference Range
Hemoglobin (g/dL)	15.1	13.7	11.5-15.5
Platelets (×10^3^/µL)	386	264	150-450
Lymphocytes (%)	21.2	48.8	20-40
Neutrophils (%)	67	37.7	40-70
Eosinophils (%)	1.8	5.8	1-4
CRP (mg/L)	31.5	23.4	<5
ESR (mm/hr)	30	45	<20
Creatinine (mg/dL)	0.47	0.45	0.5-1.0
BUN (mg/dL)	8	6	7-20
AST (U/L)	24	33	10-40
ALT (U/L)	15	13	7-35
Albumin (g/dL)	4.6	-	3.5-5.0

Electrolytes (sodium, potassium, chloride, bicarbonate) were within physiological range. Coagulation tests, including prothrombin time (PT) and international normalized ratio (INR), were normal. Complement C3 and C4, immunoglobulins, rheumatoid factor, and anti-dsDNA antibodies were ordered to rule out an immune-mediated or autoimmune etiology of the progressively evolving mucocutaneous lesions, and all were normal. Urinalysis was negative for hematuria, proteinuria, and infection, and blood cultures remained negative. A broad viral and atypical bacterial culture panel, including *M. pneumoniae*, *Klebsiella*, and Epstein-Barr virus (EBV), was all negative, and the nasopharyngeal swab was negative for significant organisms.

The patient was started on intravenous acyclovir, given the recent history of varicella infection and concern for persistent or complex viral mucocutaneous disease. Additionally, the patient was started on empirical antibiotics with ceftazidime and azithromycin, the latter to cover for possible atypical infection. A single dose of intravenous hydrocortisone and antihistamines, including cetirizine, was also initiated. His skin and mucosal lesions continued to progress. Around days 5-6 of admission, after consultation with the immunology and allergy team, with consideration of the continued severe mucocutaneous involvement, poor response to antibiotics, and the presence of tense blisters with oral involvement, there was a change in the working diagnosis to EMM versus SJS. The patient was started on oral methylprednisolone and topical clobetasol propionate with a gradual response.

The patient had been treated with intravenous immunoglobulin (IVIG) 40 mg over eight hours starting around day 9 of admission, and there was a gradual resolution and improvement of skin and mucosal lesions. The patient was discharged after 10 days of hospitalization, clinically improved, and stable. She was counseled to avoid NSAIDs. At follow-up one week after discharge, there was a dramatic improvement in cutaneous lesions and improvement in oral mucosa healing. There were no new lesions. The skin lesions were markedly improved when compared to those during initial hospitalization, when the patient had diffuse target lesions and tense bullae. There was complete resolution of blistering, and active inflammation was not present. The lesions were now post-inflammatory hyperpigmented patches.

**Figure 3:**
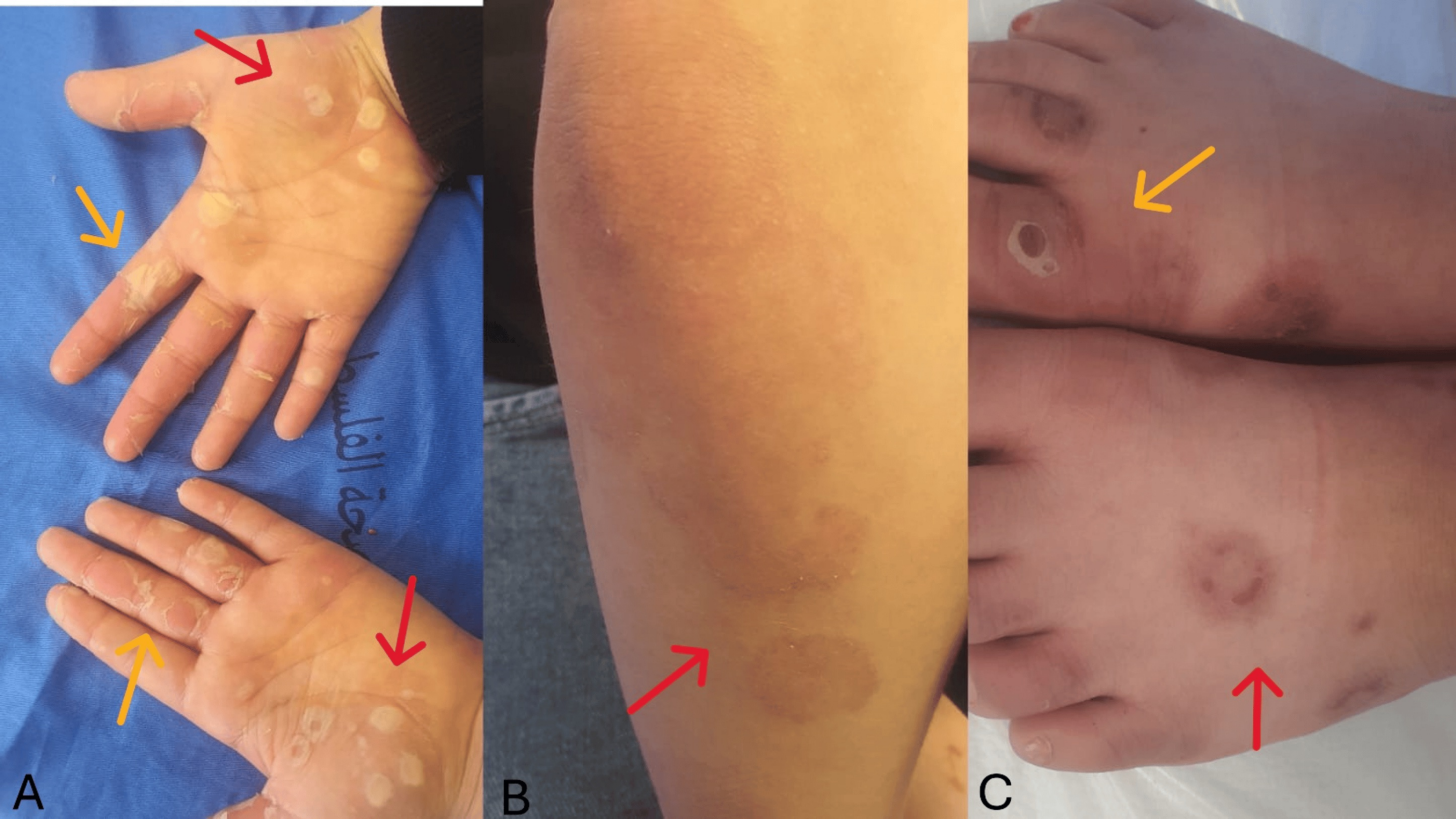
Dorsal foot, palmar, and elbow findings showing cutaneous resolution marked by hyperpigmented macules and absence of erythema or blistering. (A) Yellow arrows indicate multiple small, well-defined palmar vesicular/erythematous lesions with superficial desquamation mostly over the fingers and palm, and red arrows indicate multiple larger annular lesions with central clearing over the palm. (B) The red arrow indicates a solitary, well-defined, round hyperpigmented plaque on the lower limb, consistent with resolving or post-inflammatory changes. (C) The yellow arrow indicates a circular lesion over the dorsum of the foot with central erosion and a surrounding pale rim; red arrows indicate multiple targetoid lesions over the dorsal foot with central dusky areas and peripheral erythema.

On examination, the dorsal aspect of the foot showed well-defined circular hyperpigmented macules with residual targetoid appearance, but without erythema, crusting, or active lesions (Figure 3A). The palmar surfaces demonstrated complete resolution of the previously noted bullae, with only faint hyperpigmented areas and mild superficial peeling over the fingers, consistent with re-epithelialization (Figure 3B). There were residual hyperpigmented patches with ill-defined margins and a subtle targetoid pattern on the extensor surface of the elbow without active erythema, blistering, or crusting, consistent with post-inflammatory changes (Figure 3C). Overall, the skin was stable and had improved significantly. Based on the clinical improvement, oral and topical corticosteroids were stopped, and treatment with topical Moist Exposed Burn Ointment (MEBO) was continued. The patient was scheduled for a second follow-up visit in one week to repeat the examination and to confirm complete resolution.

## Discussion

While infections are more common in pediatric populations, SJS is a rare but severe mucocutaneous hypersensitivity reaction that is more often drug-induced [8]. Approximately 90% of cases of SJS/TEN are drug-related, while only around 6% are infectious (e.g., *M. pneumoniae*, herpesviruses), as shown in a large systematic review by Wang et al. that included more than 1,000 cases [9]. In order to differentiate infection-driven instances from classical drug-induced SJS/TEN, the term reactive infectious mucocutaneous eruption (RIME) was introduced due to the disparity in pediatric trigger patterns [8].

The underlying pathophysiology involves a T-cell-mediated immune response, predominantly driven by CD8+ cytotoxic lymphocytes, with an additional contribution from dysregulated T helper cells [5]. The subsequent immunological activation causes the release of cytotoxic mediators that cause keratinocyte apoptosis and epidermal necrosis. Proinflammatory cytokines, especially interleukin-15 (IL-15), stimulate T-cell and natural killer (NK) cell activity to further augment immune responses, fueling the inflammatory cascade to elicit the characteristic clinical picture shown in our patient with fever, mucosal involvement, and generalized rash [5].

Drug exposure remains the primary and well-established trigger in most cases, particularly antibiotics, anticonvulsants, analgesics, and antineoplastic agents [9]. In our patient, no high-risk medications classically associated with SJS were identified; however, intermittent exposure to ibuprofen was documented during the early phase of varicella infection. Although the exposure was limited, NSAIDs have been reported as possible triggers of SJS in several studies [10-12], with a recent case reported even after a single therapeutic dose of ibuprofen [13]. This example highlights a potential risk associated with commonly used over-the-counter drugs and the unpredictable, idiosyncratic nature of this hypersensitive reaction. Importantly, minimal exposure to such agents, especially during viral infections like varicella, is capable of increasing susceptibility by decreasing the threshold for drug-associated hypersensitivity episodes. In general, the time relationship between exposure to ibuprofen and onset of symptoms suggests a potential drug-associated factor, which, together with simultaneous viral infection, may result in a multifactorial etiology with synergistic immune activation. Accordingly, a drug-associated factor cannot be excluded.

A real-world analysis of the FDA Adverse Event Reporting System (FAERS) database identified NSAIDs as rare but relevant causes of SJS with a total of 1,868 reported cases from 2004 to 2021. Of these agents, ibuprofen had the highest disproportionality signal and the largest number of reported cases, typically presenting with a short median onset of three days [12]. The onset in our patient was delayed by approximately 15 days, which is atypical compared to most ibuprofen-associated cases. Even though ibuprofen-related SJS is accompanied by high rates of hospitalization, its mortality seems lower than that of aspirin or diclofenac [12].

Moreover, the etiology of SJS may also be influenced by genetic predisposition. The concept of a genetic predisposition to disease, even with similar drug exposure, has been supported by the identification that some human leukocyte antigen (HLA) alleles are associated with an increased risk of severe cutaneous adverse drug reactions [14]. This may help explain how our patient developed SJS with intermittent and minimal exposure to ibuprofen.

Due to the lack of standardised diagnostic criteria, the diagnosis of SJS/TEN is primarily clinical and is typically suspected in patients who present with fever, flu-like symptoms, and acute mucocutaneous lesions after recent exposure to a potentially offending drug, usually within one to four weeks [9]. Direct immunofluorescence is typically negative, and skin biopsies may show keratinocyte death with a T-cell-mediated inflammatory infiltrate, but they are not specific [15]. The diagnosis in this case was made clinically based on characteristic findings and temporal association with drug exposure and recent varicella infection; laboratory tests were conducted to rule out differential diagnoses, assess systemic involvement, and evaluate complications, so no skin biopsy was performed in our case.

Diagnostic ambiguity is often reported in the literature, with overlapping features with sepsis, allergic reactions, and infectious exanthems causing delays [6,16,17]. We witnessed this difficulty in our patient, who initially was considered to have a secondary bacterial infection superimposed on varicella. However, a diagnosis within the spectrum of erythema multiform major versus SJS was supported by the following development of characteristic target lesions, mucosal involvement, and blistering.

Multiple systems may be involved in long-term SJS complications. Ocular sequelae may include dry eye and symblepharon, while cutaneous sequelae may include scarring, pigmentary changes, and persistent pruritus. Respiratory complications, including bronchiolitis, bronchiectasis, and chronic bronchitis, have also been reported [15]. Acute kidney injury, hydronephrosis, respiratory failure, obstructive uropathy, secondary infections, fluid and electrolytes disturbances, and long-term eye damage can occur in severe cases [6,11,18,19]. Interestingly, none of these serious problems occurred in our patient, indicating a very mild systemic course.

While systemic therapies like corticosteroids, IVIG, and cyclosporine may be taken into consideration in certain cases based on the severity of the disease, management strategies documented in the literature emphasize supportive care and prompt discontinuation of the suspected trigger [15]. Following the initiation of systemic corticosteroids, our patient’s condition dramatically improved, and after IVIG, it was optimally stabilized. In contrast to many documented cases when early ophthalmologic intervention is frequently necessary, the patient did not experience significant ocular sequelae and merely needed close observation since she had only mild eye irritation [6,16,19].

A key distinguishing feature of this case is the temporal relationship with recent varicella infection, with delayed onset of mucocutaneous manifestations approximately 20 days later. Infection-related SJS is a well-described entity, but post-varicella presentations are rare in the literature. Furthermore, the patient's significant presence of arthralgia with functional limitation, extensive palmoplantar involvement, and classic target lesions makes for an atypical clinical picture.

## Conclusions

This case highlights the multifactorial etiology of SJS in children and the need for heightened vigilance for NSAID-induced SJS in patients who have also had a recent viral infection. The patient was treated with intermittent low-dose ibuprofen, which may have been the inciting drug, as it displayed a temporal relationship with the onset of symptoms, and this report also contributes to the growing body of evidence that very low levels of drug exposure can cause SJS in patients who are susceptible. Additionally, the patient had a concomitant varicella-zoster virus infection, which may have further contributed to immune dysregulation and lowered the threshold for the development of SJS. Early diagnosis of the patient was difficult because of overlap with infectious exanthems. However, the diagnosis was later confirmed by target lesions, mucosal involvement, and palmoplantar rash. Fortunately, this patient had no discernible systemic complications and responded well to corticosteroids and IVIG.
